# Menstrual symptoms and subjective well-being among postmenarchal adolescents

**DOI:** 10.1016/j.xagr.2023.100304

**Published:** 2023-12-26

**Authors:** Pietro Gambadauro, Gergö Hadlaczky, Danuta Wasserman, Vladimir Carli

**Affiliations:** 1 Department of Learning, Informatics, Management and Ethics, Karolinska Institutet, Stockholm, Sweden (Drs Gambadauro, Hadlaczky, Wasserman, and Carli); 2 Department of Women's and Children's Health, Uppsala University, Uppsala, Sweden (Dr Gambadauro); 3 Stockholm Health Care Services, Stockholm, Sweden (Drs Gambadauro, Hadlaczky, and Carli); 4 Res Medica Sweden, Uppsala, Sweden (Dr Gambadauro).

**Keywords:** adolescent health, menstrual health, menstruation disturbances, WHO-5, women's health

## Abstract

**BACKGROUND:**

Menstrual symptoms are predominantly studied among adults but may occur directly after menarche. Adolescent menstrual healthcare, however, faces specific obstacles and more research into menstrual symptoms as a determinant of adolescent well-being is therefore advocated.

**OBJECTIVE:**

This study aimed to investigate menstrual symptoms and their impact on everyday life and well-being among postmenarchal adolescents.

**STUDY DESIGN:**

A survey was delivered to a random sample of 1644 schoolgirls, drawn from a population-based project involving 116 lower secondary education schools (7th and 8th grade) in Stockholm, Sweden. Menstrual symptoms (ie, dysmenorrhea, heavy bleeding, irregular periods, mood disturbance, other general symptoms) were investigated through multiple choice questions and defined according to their impact on everyday life as mild (seldom affected), moderate (affected but possible to cope) and severe (affected and difficult to cope). Subjective well-being was measured with the World Health Organization Five Well-Being index. Postmenarchal respondents were eligible for analysis; those with incomplete outcome data or using hormonal contraception were excluded. The frequency and severity of symptoms across different postmenarchal years (1st, 2nd, 3rd, 4th, or 5th+ year after menarche) were studied with Chi-square and Kendall's tau statistics. Analysis of variance was used to study the association between menstrual symptoms and World Health Organization Five Well-Being index scores. A composite menstrual health index variable was obtained through principal component analysis and used to study the overall impact of menstrual symptoms on well-being in regression analyses.

**RESULTS:**

Of 1100 postmenarchal girls (mean age, 14.1±0.7 years), 93.2% reported menstrual symptoms, 81.3% had at least 1 moderate symptom and 31.3% had at least 1 severe symptom. The most frequent symptoms were dysmenorrhea (80.4%) and mood disturbance (81.1%), followed by irregular periods (67.9%), heavy bleeding (60.4%), and other general symptoms (60.4%). Throughout postmenarchal years, there was a significant increase in frequency and severity (*P*<.001) of dysmenorrhea (τ=0.148), heavy bleeding (τ=0.134), mood disturbance (τ=0.117), and other general symptoms (τ=0.110), but not irregular periods (τ=−0.0201; *P*=.434). Girls with menstrual symptoms had significantly lower World Health Organization Five Well-Being index scores than those without symptoms (mean difference, −17.3; 95% confidence interval, −22.4 to −12.3). Analysis of variance showed significant associations (*P*<.001) with World Health Organization Five Well-Being index scores for each of the examined menstrual symptoms. In post hoc pairwise comparisons with peers without symptoms, the greatest reductions in World Health Organization Five Well-Being index score were found among girls with severe symptoms (mean difference for: dysmenorrhea, −20.72; heavy bleeding, −15.75; irregular periods, −13.81; mood disturbance, −24.97; other general symptoms, −20.29), but significant differences were observed even for moderate or mild symptoms. The composite menstrual health index was significantly associated with World Health Organization Five Well-Being index scores in regression analysis, independently of age, age at menarche, body mass index, smoking, physical activity, own and parental country of birth, biparental care, and socioeconomic status.

**CONCLUSION:**

Despite growing awareness about the relevance of menstruation to women's health, unmet menstrual health needs are a potential threat to the well-being of adolescents. Education, screening, and clinical competence are important tools to reduce the burden of menstrual symptoms during adolescence and to prevent long-term consequences. The development of novel person-centered strategies should be a priority for clinical practice and research in adolescent menstrual health.

**AJOG Global Reports at a Glance**

**Why was this study conducted?**

More research into menstrual health as a determinant of adolescent well-being is being advocated. No previous data are available on the association between adolescent menstrual symptoms and the World Health Organization Five Well-Being (WHO-5) index, a valid measure of subjective well-being.

**Key findings**

In a sample of 1100 Swedish adolescents, menstrual symptoms such as dysmenorrhea, heavy bleeding, irregular periods, mood disturbance, and other general symptoms, were highly prevalent and associated with lower WHO-5 scores, even when not subjectively reported as an unmanageable burden for daily life.

**What does this add to what is known?**

Despite growing awareness about the relevance of menstruation to women's health, unmet menstrual health needs are a potential threat to adolescent well-being. Generic measures of well-being such as the WHO-5 can facilitate person-centered strategies for care and research in adolescent menstrual health.

## Introduction

Menstruation is highly relevant to women's health because most women of reproductive age menstruate and many of them experience associated symptoms such as pain, heavy bleeding, period irregularities, or mood swings at some point between menarche and menopause.^1^^,^^2^ Menstrual health has traditionally been underrepresented in research but the burden of menstrual symptoms on women's well-being is increasingly addressed in scientific and social contexts.^3^ Nevertheless, adolescent menstrual health still faces specific obstacles.^4^ Poor menstrual literacy, normalization, and stigma prevent young women from reporting or seeking help for menstrual health problems.^5^ Symptoms among younger teens may often slip through the cracks because of unclear care responsibility among gynecology, pediatrics, or primary care services.^4^^,^^6^ Furthermore, children may have a relatively weak position in healthcare systems where choice is increasingly relevant.^7^

Developments in adolescent menstrual health are also hindered by conceptual barriers. Clinical practice and research have traditionally focused on objective or quantitative outcome measures, such as blood loss volume, number of days or points on analog scales. However, there is a proven divide between symptom perception and objectively measurable outcomes.^1^ In addition, research studies usually evaluate specific menstrual symptoms and their direct consequences, but rarely address generic health and well-being measures that allow comparisons across populations, interventions and medical conditions.^8^

The mentioned challenges are consistent with global reports highlighting unmet menstrual health needs among adolescents from Africa,^9^^,^^10^ Asia,^11^, ^12^, ^13^, ^14^ Australia,^15^ Europe,^2^^,^^16^, ^17^, ^18^ North America,^19^ and South America.^20^ Such observations contravene children's right to the “enjoyment of the highest attainable standard of health”^21^ and have prompted recent calls for improvements in person-centered care and research into menstrual symptoms as an important determinant of adolescent health.^8^^,^^22^

This study aimed to evaluate the frequency and severity of menstrual symptoms, and their impact on subjective well-being, among Swedish adolescents in their early postmenarchal years.

## Materials and Methods

### Design and study population

This was a cross-sectional study of anonymized survey data from the Youth Aware of Mental health (YAM) project for mental health promotion among pupils attending lower secondary education (7^th^ and 8th grade; 13–15 years) in Stockholm, Sweden. The project was based on an internationally-effective school–based intervention for mental health,^23^ which was thereafter implemented in 116 schools located in Stockholm County by Region Stockholm and Karolinska Institutet between 2016 and 2019. A structured self-report health survey was distributed to a sample of 1644 adolescent schoolgirls, which was drawn randomly (1:3) from the baseline population of the YAM project. The survey was completed on mobile tablet devices during a single classroom session and included items regarding sociodemographic factors, behavior, well-being, and menstrual health. All postmenarchal respondents with known age at menarche were eligible for inclusion whereas those with incomplete outcome data or using hormonal contraception were excluded.

### Measurements

#### Menstrual symptoms

An ad hoc set of multiple choice questions (Appendix) investigated the presence and severity of menstrual symptoms belonging to the following 5 categories: dysmenorrhea, heavy menstrual bleeding, irregular periods, mood disturbance, and other general symptoms (eg, malaise, headache, tiredness, nausea, vomit, and diarrhea). The questionnaire was loosely based on a previous Swedish scoring system for dysmenorrhea showing good correlation with a linear analogue scale.^24^ Each symptom was defined according to its impact on everyday life with 4 possible levels: 1-never affected, 2-seldom affected, 3-affected but possible to cope, and 4-affected and difficult to cope. Levels 1 to 4 were respectively conceptualized as absent, mild, moderate, and severe symptom.^24^

#### Well-being

Subjective well-being was evaluated by means of the World Health Organization Five Well-Being Index (WHO-5), a widely used questionnaire including 5 positively-phrased items regarding feelings experienced during the previous 2 weeks.^25^ Each item is rated from 0 to 5 and the raw sum of all item scores is multiplied by 4 to obtain a percentage scale ranging from 0 (ie, complete absence of well-being) to 100 (ie, highest well-being).

#### Covariates

Age was calculated from the date of birth, whereas age at menarche was self-reported and used to calculate the postmenarchal year (ie, 1st, 2nd, 3rd, 4th, or 5th+ year after menarche). The body mass index (BMI) was calculated from self-reported height and weight, and the results were categorized, according to age-specific percentiles,^26^ as underweight (<5°), normoweight (≥5° and <85°), or overweight (≥85°). Other considered dichotomous variables were sedentary behavior (yes/no), smoking (yes/no), own country of birth (Sweden/other), parental country of birth (Sweden/other), biparental care (yes/no), lower socioeconomic status (SES) (yes/no). Sedentary behavior was defined as engaging in sport activities less frequently than once a week during the last 6 months. Biparental care was defined as living with 2 parents, either concurrently or in separate households. The SES was evaluated subjectively by asking whether the respondent had "enough money to be able to do the same things as your mates," and the answers “never,” “rarely,” and “sometimes” were categorized as lower SES.

### Statistical analysis

The frequency and severity of menstrual symptoms were studied descriptively in the overall sample. Differences in covariates among girls reporting absent, mild, moderate, or severe symptoms were tested using Chi-square or Fisher's exact tests (with expected cell frequencies <5) for categorical variables, and analysis of variance for continuous variables. Chi-square statistics were used to analyze the prevalence of each symptom in the postmenarchal years, whereas Kendall's tau statistics were used to measure the strength and direction of the relationship. One-way analysis of variance was used to study the association between each menstrual symptom and WHO-5 scores. Post hoc pairwise comparisons of WHO-5 scores between different symptom severity groups were summarized as mean differences (MDs) and 95% confidence intervals (CIs). To study the overall effect of menstrual symptoms on well-being, a composite index variable was obtained through principal component analysis, based on polychoric correlations between menstrual symptom variables. The first principal component (eigenvalue=2.81; 56% of variance) was used as standardized menstrual health index, with higher values representing less symptoms and therefore, higher menstrual health (ie, being desirable). Linear regression was then used to examine the association between the menstrual health index and WHO-5 scores, respectively as independent and dependent variable, and accounting for age and age at menarche. Further analyses were adjusted for BMI, smoking, sedentary behavior, own and parental country of birth, biparental care, and SES. Statistical significance was defined as a *P* value of <.05. The statistical analyses were performed in R (R Foundation for Statistical Computing, Vienna, Austria) with RStudio (Posit Software, PBC, Boston, MA) for macOS (Apple Inc., Cupertino, CA).^27^^,^^28^

### Informed consent and ethical approval

Participation in the YAM project was voluntary and informed consent was obtained from all participants before inclusion. According to local requirements, additional informed consent was obtained by the legal caregivers of pupils younger than 15 years old. Ethical approval was received from the Regional Ethics Committee in Stockholm (Regionala etikprövningsnämnden i Stockholm; ref. 2015/2175-31/5).

## Results

**Figure 1:**
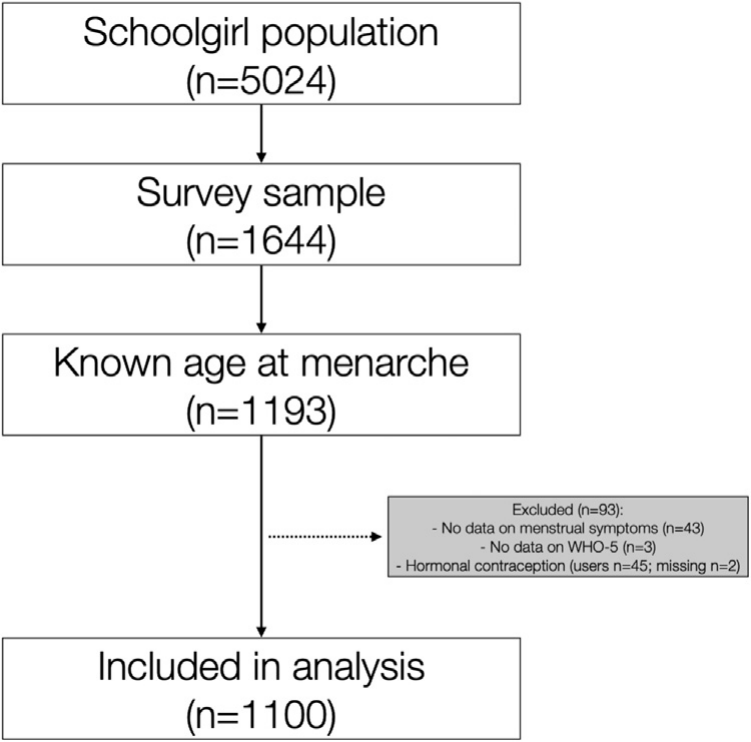
Study flowchart

Age at menarche was reported by 1193 girls who were therefore eligible for the study. After excluding 93 girls due to lack of outcome data or hormonal contraception, 1100 were eventually included in analysis (Figure 1). The mean age was 14.1 years (standard deviation [SD], 0.7), whereas the mean age at menarche was 12.2 years (SD, 1.0). The mean BMI was 19.7 (SD 2.7), and the frequencies in the underweight, normoweight, and overweight categories were 4.8%, 83.0%, and 12.2%, respectively. One-quarter of respondents (24.6%) were categorized as sedentary, whereas 4.8% reported smoking. Sweden was the country of birth to most respondents (90.8%) as well as to at least one of their parents (84.1%). Biparental care was reported by 89.2%, whereas 14.5% had a lower perceived SES.

Gambadauro. Adolescent menstrual health and subjective well-being. Am J Obstet Gynecol Glob Rep 2024.

**Figure 2:**
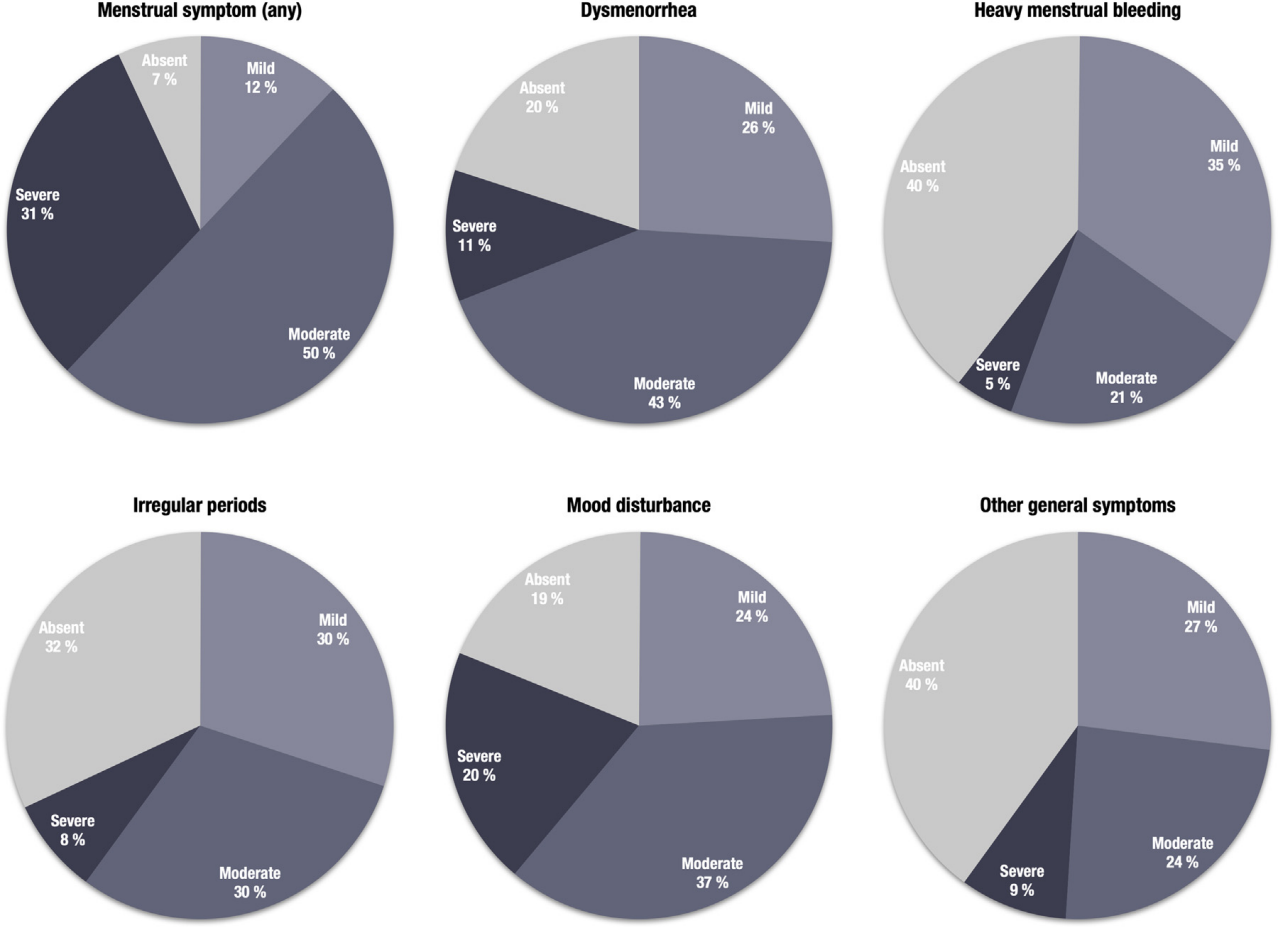
Menstrual symptoms among 1100 Swedish adolescents Figure shows *pie charts* displaying the frequency and severity of menstrual symptoms (rounded %).

**Figure 3:**
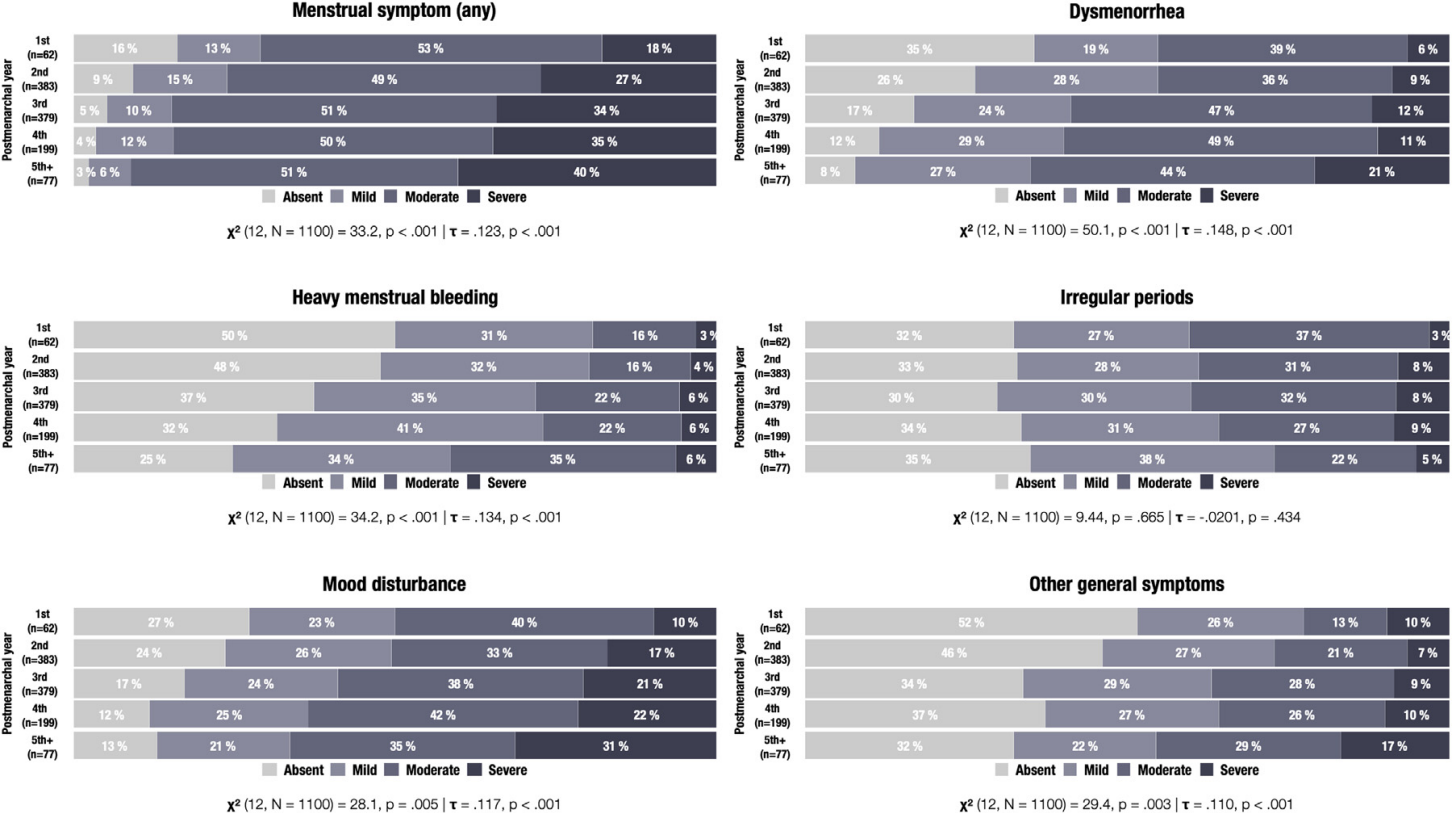
Menstrual symptoms during early postmenarchal years Figure shows *bar charts* displaying the frequency of symptoms (rounded %) and analyses of the association between symptoms and postmenarchal years with Chi-square (χ^2^) and Kendall's tau (τ) statistics.

Overall, 93.2% reported at least 1 menstrual symptom, 81.3% had at least 1 moderate symptom, and 31.3% had at least 1 severe symptom (Figure 2). Lower age at menarche, smoking, parental country of birth (Sweden), and lower SES were statistically associated with menstrual symptom reporting whereas no significant associations were found for age, BMI, sedentary behavior, own country of birth, and biparental care (Table 1). The most reported symptoms were dysmenorrhea (80.4%) and mood disturbance (81.1%), followed by irregular periods (67.9%), heavy bleeding (60.4%), and other general symptoms (60.4%) (Figure 2). Throughout postmenarchal years, there was a significant increase in prevalence and severity of dysmenorrhea, heavy bleeding, mood disturbance, general symptoms, but not of irregular periods (Figure 3).

Gambadauro. Adolescent menstrual health and subjective well-being. Am J Obstet Gynecol Glob Rep 2024.

**Table 1:** Sample characteristics and menstrual symptom reporting.

Characteristics		Total sample^a^	Menstrual symptom(s)^a^				
			Absent	Mild	Moderate	Severe	
		N=1100	n=75	n=131	n=550	n=344	*P* value^b^
Age	Mean (SD)	14.1 (0.7)	13.9 (0.7)	14.0 (0.8)	14.1 (0.7)	14.0 (0.7)	.064
Age at menarche	Mean (SD)	12.2 (1.0)	12.5 (0.8)	12.3 (0.9)	12.2 (1.0)	12.0 (0.9)	<.001
BMI category	Underweight	50 (4.8%)	4 (5.9%)	7 (5.5%)	25 (4.8%)	14 (4.3%)	.18
	Normoweight	869 (83.0%)	61 (89.7%)	107 (83.6%)	441 (83.8%)	260 (80.0%)	
	Overweight	128 (12.2%)	3 (4.4%)	14 (10.9%)	60 (11.4%)	51 (15.7%)	
	missing	53	7	3	24	19	
Sedentary behavior	No	827 (75.4%)	57 (79.2%)	104 (79.4%)	423 (76.9%)	243 (70.6%)	.088
	Yes	270 (24.6%)	15 (20.8%)	27 (20.6%)	127 (23.1%)	101 (29.4%)	
	missing	3	3	0	0	0	
Smoking	No	1047 (95.2%)	74 (98.7%)	129 (98.5%)	526 (95.6%)	318 (92.4%)	.014
	Yes	53 (4.8%)	1 (1.3%)	2 (1.5%)	24 (4.4%)	26 (7.6%)	
Born in Sweden	No	101 (9.2%)	10 (13.3%)	16 (12.3%)	44 (8.0%)	31 (9.0%)	.26
	Yes	996 (90.8%)	65 (86.7%)	114 (87.7%)	505 (92.0%)	312 (91.0%)	
	missing	3	0	1	1	1	
Parent(s) born in Sweden	No	174 (15.9%)	15 (20.0%)	30 (23.1%)	70 (12.8%)	59 (17.4%)	.016
	Yes	917 (84.1%)	60 (80.0%)	100 (76.9%)	476 (87.2%)	281 (82.6%)	
	missing	9	0	1	4	4	
Biparental care	No	119 (10.8%)	14 (18.7%)	11 (8.4%)	52 (9.5%)	42 (12.2%)	.062
	Yes	981 (89.2%)	61 (81.3%)	120 (91.6%)	498 (90.5%)	302 (87.8%)	
Lower socioeconomic status	No	938 (85.5%)	70 (94.6%)	115 (88.5%)	480 (87.3%)	273 (79.6%)	<.001
	Yes	159 (14.5%)	4 (5.4%)	15 (11.5%)	70 (12.7%)	70 (20.4%)	
	missing	3	1	1	0	1	

*BMI,* body mass index; *SD,* standard deviation

a Mean (SD) or number (percentage)

b Chi-square or Fisher exact test (categorical variables) and one-way analysis of variance (continuous variables).

Gambadauro. Adolescent menstrual health and subjective well-being. Am J Obstet Gynecol Glob Rep 2024.

**Figure 4:**
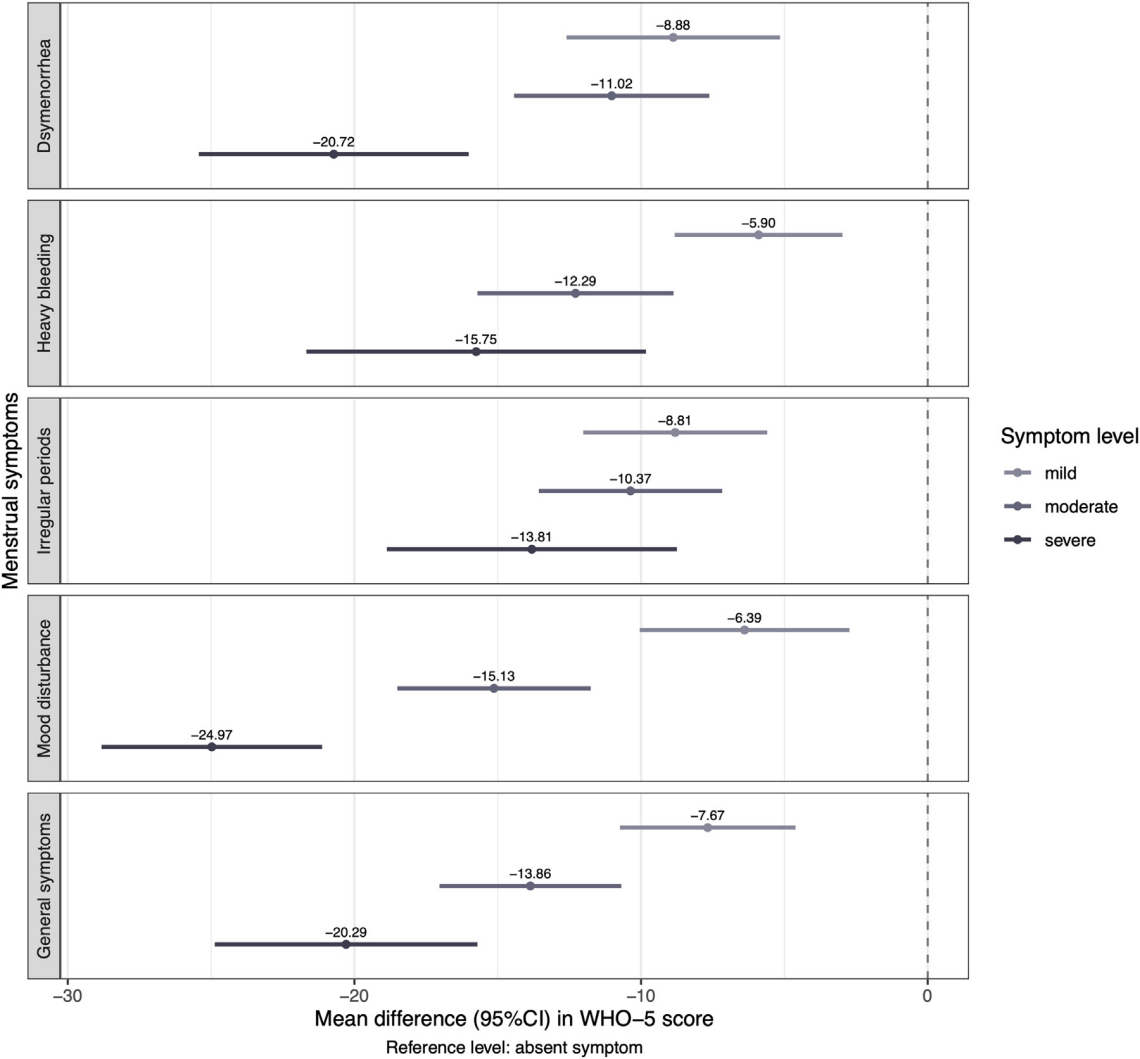
WHO-5 score differences between girls with and without menstrual symptoms Figure shows mean differences (with 95% confidence intervals) in WHO-5 scores derived from post hoc pairwise comparisons between girls with a mild, moderate, or severe menstrual symptom, and girls without the symptom.

The mean WHO-5 score in the overall sample was 58.7 (SD, 21.9; 95% CI, 57.4–60.0). Girls with menstrual symptoms had significantly reduced WHO-5 scores when compared with those without (MD, −17.3; 95% CI, −22.4 to −12.3). Analysis of variance showed a significant effect (*P*<.001) on WHO-5 scores for each of the symptoms examined (Table 2). In post hoc analyses, the MDs in WHO-5 score were largest among girls with severe symptoms, but statistically significant even for those with moderate and mild symptoms, compared with girls without the symptoms (Figure 4). The composite menstrual health index variable was significantly associated with WHO-5 scores in regression analyses, adjusted for the effects of age, age at menarche, BMI, smoking, sedentary behavior, own and parental country of birth, biparental care, and SES (Table 3).

**Table 2:** Association between menstrual symptoms and WHO-5 scores among 1100 Swedish adolescents

Menstrual symptom	Dependent variable: WHO-5 score				
	F_Fisher_	df1	df2	*P* value	$\omega_{p}^{2}$ (95% CI)
Dysmenorrhea	26.8	3	1096	<.001	0.066 (0.039–0.094)
Heavy menstrual bleeding	21.8	3	1096	<.001	0.054 (0.029–0.080)
Irregular periods	19.1	3	1096	<.001	0.047 (0.024–0.072)
Mood disturbance	64.4	3	1096	<.001	0.147 (0.110–0.184)
Other general symptom	39.4	3	1096	<.001	0.095 (0.063–0.130)

Associations between each menstrual symptom variable (in 4 levels: absent, mild, moderate, and severe) and WHO-5 well-being scores, studied with one-way analysis of variance with Fisher F-test statistics and the $\omega_{p}^{2}$ effect size.

*WHO-5*, World Health Organization Five Well-Being index.

Gambadauro. Adolescent menstrual health and subjective well-being. Am J Obstet Gynecol Glob Rep 2024.

**Table 3:** Menstrual health as a predictor of well-being among adolescents

Characteristics	Dependent variable: WHO-5 score		
Predictors	Model 1	Model 2	Model 3
	Coefficient (95% CI)	Coefficient (95% CI)	Coefficient (95% CI)
Intercept^a^	58.73^b^ (57.55–59.92)	62.24^b^ (60.78–63.70)	63.02^b^ (61.45–64.58)
Menstrual Health index^c^	8.22^b^ (7.07–9.36)	7.49^b^ (6.33–8.65)	7.05^b^ (5.90–8.20)
Age	−0.87 (−2.61 to 0.87)	−0.48 (−2.23 to 1.27)	−0.48 (−2.22 to 1.26)
Age at menarche	−0.61 (−1.90 to 0.68)	−1.07 (−2.37 to 0.24)	−0.59 (−1.90 to 0.73)
Underweight	—	−8.07^d^ (−13.68 to −2.45)	−7.15^e^ (−12.65 to −1.65)
Overweight	—	−4.93^d^ (−8.62 to −1.25)	−4.13^e^ (−7.80 to −0.45)
Sedentary behavior	—	−7.22^b^ (−10.03 to −4.41)	−6.62^b^ (−9.44 to −3.81)
Smoking	—	−11.81^b^ (−17.33 to −6.28)	−11.22^b^ (−16.69 to −5.76)
Born outside Sweden	—	—	0.30 (−4.07 to 4.67)
Parents born outside Sweden	—	—	4.35^e^ (0.86–7.84)
Nonbiparental care	—	—	−2.89 (−6.78 to 1.00)
Lower perceived socioeconomic status	—	—	−10.73^b^ (−14.14 to −7.32)
Observations	1100	1045	1032

Estimates of the association between the composite Menstrual Health index variable, as predictor, and WHO-5 well-being scores, as dependent variable. The coefficients are derived from 3 multivariable linear models, accounting for the effect of the following predictors:

(1) Menstrual Health index+age (centered)+age at menarche (centered)

(2) (1)+BMI category (underweight/overweight, with normoweight as reference)+sedentary behavior+smoking

(3) (2)+Born outside Sweden+parents born outside Sweden+nonbiparental care+lower socioeconomic status.

*BMI,* body mass index; *CI,* confidence interval; *WHO-5*, World Health Organization Five Well-Being index

a Reference level

b *P*<.001

c Composite variable obtained from the items of the menstrual symptom questionnaire using principal component analysis (see text)

d *P*<.01

e *P*<.05.

## Comment

### Principal findings

This study evaluated menstrual symptoms and subjective well-being in a random, population-based sample of 1100 adolescent schoolgirls in Stockholm, Sweden. Overall, 9 out of 10 girls had menstrual symptoms, 4 out of 5 reported related interference with daily life, and a third of the sample reported severe symptoms they could not cope with. Dysmenorrhea and mood disturbance were reported more frequently, but irregular periods, heavy bleeding and other general symptoms were also highly prevalent. The frequency and severity of dysmenorrhea, heavy bleeding, mood changes, and general symptoms increased significantly through the postmenarchal years, whereas irregular periods remained stable. The WHO-5 well-being scores of girls with menstrual symptoms were significantly lower than those of girls without symptoms. Interestingly, the reduction in well-being was greatest among girls with severe symptoms but significant for moderate and mild symptoms as well. Overall menstrual health was significantly associated with WHO-5 scores independently of age, age at menarche, and several other covariates.

### Results in the context of what is known

This study shows that menstrual symptoms are extremely common among Swedish teenagers, a third of whom have their daily life affected during menstruation to such an extent that they cannot cope. Young postmenarchal girls are therefore no exception to the high prevalence of menstrual symptoms observed among population-based samples of women of reproductive age.^2^ This finding, along with similar reports from different countries and across all continents,^9^, ^10^, ^11^, ^12^, ^13^, ^14^, ^15^, ^16^, ^17^, ^18^, ^19^, ^20^ defines adolescent menstrual symptoms as a serious and global public health issue.

Interpretation of prevalence data from adolescent menstrual health studies should consider methodological issues, such as the timing of observation in relation to age and age of menarche. Adolescents are often treated as a homogeneous group, but adolescence is a period of intense change, and the pattern of menstrual symptoms varies significantly in the postmenarchal years. For example, whereas irregular periods were consistently frequent in the present study, the prevalence of other severe symptoms increased 2 to 3 times within a few years of menarche. These variations have specific biological explanations. Irregular periods after menarche are consistent with the immaturity of the female reproductive axis and the associated anovulatory cycles, whereas the tendency for symptoms such as menstrual pain or mood changes to increase is related to the progressive development of mature ovulatory cycles.^29^

Methods for measuring symptoms, local norms, and cultural perceptions should also be considered. Our study highlights individual views on the impact of symptoms on daily life, whereas clinical practice and research have traditionally been based on objective or quantitative outcome measures.^1^ The latter has been defined as a conceptual barrier to understanding and improving menstrual health because there is a divide between how symptoms are perceived and quantitative measurements.^1^

The relationship between menstrual symptoms and well-being in our study is consistent with recent observations on reduced health-related quality of life^30^ and highlights the need to study person-reported outcomes.^1^^,^^31^ As expected, symptoms classified as severe in the study had the greatest impact on WHO-5 well-being scores. This confirms previous findings that adolescents receiving specialist care for menstrual problems have significantly reduced health-related quality of life.^32^ However, even symptoms that were not subjectively reported as an unmanageable burden were significantly associated with reduced well-being. The apparent inconsistency can be explained by lasting sociocultural barriers such as normalization or stigma toward menstruation and related symptoms, which hinder menstrual health care in low-, middle-, and high-income countries.^5^

### Clinical implications

The findings of this study suggest an urgent need for people-centered strategies^33^ that involve improved education, prevention, and care in menstrual health. Widespread menstrual literacy is essential to empower girls with symptoms and to counter normalization or stigma.^1^ Organized screening in the context of preventive healthcare would require planned and structured assessments of menstrual health. Opportunistic screening could also prove useful, consistently with recent calls to consider the menstrual cycle as a vital sign in the context of general adolescent healthcare.^22^ Both organized and opportunistic screening can effectively be initiated within community-based or school-based healthcare^30^; however, professional development and networking with trained specialists are warranted because of the inherent limitations of primary care.^34^, ^35^, ^36^, ^37^ Planning and timing of educational and screening strategies should consider the value of anticipatory guidance, aimed at children and caretakers even before the typical age of menarche.^22^

Those with persistent menstrual symptoms should be attended to with specific expertise in adolescent gynecology^36^^,^^37^ and awareness of the potential impact on psychological well-being. In this regard, it should be noted that the WHO-5 index has adequate validity for the screening of depression.^25^ Attention should be paid to psychosocial factors because of the important role of individual vulnerabilities and the social context as determinants of adolescent health.^4^ Furthermore, serious and chronic conditions cause nonspecific symptoms such as pain (eg, endometriosis), heavy bleeding (eg, coagulation disorders), period irregularities (eg, polycystic ovary syndrome or eating disorders), or mood swings (eg, premenstrual syndrome). Such conditions place an additional burden on well-being but can be overlooked and underdiagnosed. Pain and mood disturbance were indeed particularly prevalent in our study, consistently with data from the general female population.^2^ Pain during menstruation most commonly occurs due to an inflammatory response mediated by prostaglandins and leukotrienes in the absence of disease (primary dysmenorrhea), but may be associated with pelvic abnormalities such as endometriosis (secondary dysmenorrhea).^29^ The estimated prevalence of endometriosis in adolescents with dysmenorrhea is indeed high^38^; however, the condition is often diagnosed during adulthood and management delays augment its impact on physical, mental, and social health.^38^, ^39^, ^40^, ^41^ Therefore, specific strategies are needed to rule out endometriosis as the cause of adolescent dysmenorrhea, with consideration for noninvasive diagnostics and differential diagnoses (eg, infections, adenomyosis, or congenital anomalies).^42^ Similarly, mood swings, irritability, or anxiety are common among teenagers; however, in relation to the menstrual period, they can indicate premenstrual syndrome or premenstrual dysphoric disorder, and thus require specific management.^43^

### Research implications

Given that Swedish children might share health and socioeconomic conditions specific to a high-income European country, it would be useful to replicate these findings internationally. Long-term longitudinal studies could identify pathways connecting menstrual symptoms, their correlates during adolescence, and delayed health outcomes. The impact of earlier menarche among girls with menstrual symptoms should also be addressed. In our study, the difference in age at menarche between girls with and without symptoms was significant but small, and it has not been proven whether early menarche directly increases the risk.^44^ Nevertheless, being exposed to menstrual symptoms for a longer time and from a more vulnerable age could contribute to worse outcomes.

Future studies could frame menstrual health within the broader context of female reproductive health due to the biological and sociocultural links that exist between the 2 domains.^45^ For example, it would be relevant to study how menstrual health relates to developing sexuality, another important correlate of adolescent health and psychological well-being.^46^^,^^47^ Similarly, specific research could target behaviors (eg, tobacco, drugs, or alcohol consumption^46^) as signs of inadequate adjustment to physical symptoms and further threats to well-being. Additional insights may come from covariates or potential confounders whose investigation requires complementing self-report with additional data sources (eg, comorbidities, actual SES, or social support). Information related to clinical prescription or effect of specific medications (eg, analgesics or antidepressants) or healthcare contacts (eg, specialist referrals or consultations) could also help further classify symptom severity and assess current adolescent menstrual health management strategies, which are often considered inadequate.^6^

Finally, screening and treatment strategies for adolescent menstrual health should be evaluated in interventional studies targeting person-relevant outcomes. Recent international projects promote the identification of core outcomes sets in women's health research.^48^, ^49^, ^50^ No projects are yet available for adolescent menstrual health,^51^ but we believe that generic well-being measures would be useful in future trials.

### Strengths and limitations

The large study sample was randomly drawn from a population-based project, and outcome data availability among eligible girls exceeded 90%. The WHO-5 well-being index has proven to be a valid outcome measure in clinical trials and across different study fields,^25^ but to the best of our knowledge, it has not been used in adolescent menstrual health research before. The frequency and severity of a range of menstrual symptoms were analyzed during the early postmenarchal years. Furthermore, a menstrual health index was studied as a composite correlate of well-being accounting for several covariates.

The study also has limitations to consider. Cross-sectional data hinder inferences regarding causality or directionality. The mental-health-promoting initiative was implemented for all pupils, but those participating in the survey may have been different from nonparticipants (ie, volunteer bias) and information regarding the latter group is unavailable. Age at menarche was expressed in years, thus not allowing a more detailed definition of the gynecological age. Menstrual health data were not clinically ascertained but obtained through an ad hoc self-report instrument. Thus, the findings reflect subjective perceptions of the impact of menstrual symptoms rather than diagnoses. For example, specific irregularities (eg, oligomenorrhea or amenorrhea) or mood disorders (eg, premenstrual syndrome or dysphoric disorder) could not be defined. In the context of this study, however, this limitation seemed a reasonable trade-off in favor of a large population-based sample.

## Conclusions

Despite growing awareness about the relevance of menstrual health on women's well-being, this study highlights several unmet needs among Swedish adolescents. Highly prevalent menstrual symptoms during early postmenarchal years threaten adolescent health, possibly even when they are not perceived or reported as severe. Education, screening, and clinical competence are important tools to reduce the burden of menstrual symptoms during adolescence and to prevent long-term consequences. The development of person-centered strategies should be a priority for clinical practice and research in adolescent menstrual health.

## CRediT authorship contribution statement

**Pietro Gambadauro:** Conceptualization, Formal analysis, Investigation, Methodology, Resources, Writing – original draft, Writing – review & editing. **Gergö Hadlaczky:** Investigation, Methodology, Resources, Writing – review & editing. **Danuta Wasserman:** Investigation, Resources, Writing – review & editing. **Vladimir Carli:** Data curation, Investigation, Methodology, Resources, Writing – review & editing.

## Appendix Supplementary materials

Image, application 1

## Footnotes

Supplementary material associated with this article can be found in the online version at doi:10.1016/j.xagr.2023.100304.
