# Supplementary material for: Menstrual symptoms and subjective well-being among postmenarchal adolescents
Source: AJOG Glob Rep. 2023 Dec 26;4(1):100304. doi: 10.1016/j.xagr.2023.100304 (PMC10830861; doi:10.1016/j.xagr.2023.100304)
Supplement: Supplementary file 1 [file mmc1.pdf]

Menstrual symptoms and subjective well-being among postmenarchal adolescents
Gambadauro P et al.

APPENDIX

Table A. 1 English translation of the menstrual symptoms questionnaire used in the study

Are you affected in your everyday life by any of the following menstrual symptoms?

	**No**	**Seldom**	**Yes, but I can cope with it**	**Yes, and I'm having a hard time coping with it**
Pain	○	○	○	○
Heavy/prolonged bleeding	○	○	○	○
Irregular/ unpredictable menstrual cycle	○	○	○	○
Bad mood	○	○	○	○
Feeling unwell or other symptoms such as headache, tiredness, nausea, vomiting or diarrhoea	○	○	○	○
